# Seeking certainty about Intolerance of Uncertainty: Addressing old and new issues through the Intolerance of Uncertainty Scale-Revised

**DOI:** 10.1371/journal.pone.0211929

**Published:** 2019-02-11

**Authors:** Gioia Bottesi, Stefano Noventa, Mark H. Freeston, Marta Ghisi

**Affiliations:** 1 Department of General Psychology, University of Padova, Padova, Italy; 2 Methods Center, University of Tübingen, Tübingen, Germany; 3 School of Psychology, Newcastle University, Newcastle upon Tyne, United Kingdom; University of Wuerzburg, GERMANY

## Abstract

Intolerance of Uncertainty is a trans-diagnostic process that spans a range of emotional disorders and it is usually measured through the Intolerance of Uncertainty Scale-12. The current study aims at investigating some issues in the assessment of Intolerance of Uncertainty (IU) through the Italian Intolerance of Uncertainty Scale-Revised, a measure adapted from the Intolerance of Uncertainty Scale-12 to assess IU across the lifespan. In particular we address the factor structure among a large community sample, measurement invariance across gender, age, and over time, together with reliability and validity of the overall scale and its subscales. The questionnaire was administered to community (*N* = 761; mean age = 35.86 ± 14.01 years) and undergraduate (*N* = 163; mean age = 21.16 ± 2.64 years) participants, together with other self-report measures assessing constructs theoretically related to IU. The application of a bifactor model shows that the Italian Intolerance of Uncertainty Scale-Revised possesses a robust general factor, thus supporting the use of the unit-weighted total score of the questionnaire as a measure of the construct. Furthermore, measurement invariance across gender, age, and over time is supported. Finally, the Italian Intolerance of Uncertainty Scale-Revised appears to possess adequate reliability and validity. These findings support the unidimensionality of the measure, a conceptually reasonable result in line with the trans-diagnostic nature of Intolerance of Uncertainty. In addition, this study and comparison with published factor structures of the Intolerance of Uncertainty Scale-12 and of the Intolerance of Uncertainty Scale-Revised identify some issues for the internal structure of the measure. In particular, concern is expressed for the Prospective IU subscale. In light of the promising psychometric properties, the use of the Italian Intolerance of Uncertainty Scale-Revised as a univocal measure is encouraged in both research and clinical practice.

## Introduction

Intolerance of Uncertainty (IU) is the dispositional inability of an individual to tolerate the aversive reactions triggered by a perceived lack of sufficient/salient information and maintained by the related perception of uncertainty [1]. People with high levels of IU find uncertain future events as threatening, upsetting, and undesirable. In the attempt to control or avoid uncertainty, they usually endorse negative beliefs about their ability to cope with it, they experience high levels of distress, and they are likely to perform maladaptive behaviors like excessive information seeking, avoidance, or impulsive decision-making [2–6]. IU shares similarities with other psychological constructs (such as intolerance of ambiguity [7], distress tolerance [8], stress vulnerability [9], action vs. state orientation [10], indecisiveness [11], need for cognitive closure [12]), but its distinctiveness has been demonstrated [13–15] to the extent that fearing the unknown is posited to represent a fundamental fear [1]. Although IU was originally conceptualized as a cognitive vulnerability factor for worry, i.e. the core feature of Generalized Anxiety Disorder (GAD) [16, 17], it is currently considered a trans-diagnostic factor that putatively underlies neuroticism [1] and that spans a broad range of emotional disorders like Obsessive-Compulsive Disorder (OCD), social anxiety, panic disorder and agoraphobia, depression, post-traumatic stress disorder, and eating disorders [18– 21]. Additionally, recent evidence supports the notion of IU as a trans-diagnostic and trans-therapy change process. In particular, some studies demonstrated the effectiveness of unified treatment protocols focusing on IU when targeting multiple psychopathologies [22, 23]. In light of the relevance of IU as a clinical concept, the availability of a valid measure capable of reliably measuring it in both clinical and research settings appears crucial.

### The Intolerance of Uncertainty Scale

Since 1994, the Intolerance of Uncertainty Scale (IUS) [17] has represented the most widely adopted standard measure of IU [24]. The original IUS consisted of 27 items developed from the clinical observation of people suffering from GAD. Given the broad definition of IU underlying the IUS, and the inevitable dependence of factor analytic solutions on sample composition, studies designed to examine its factor structure have led to inconsistent findings, revealing either one- [25], two- [26–28], three- [29], four- [2, 30], or even five-factor solutions [17]. Most of all, factors were difficult to interpret and showed cross-loaded items [24, 31]. In particular, results suggested that the IUS lacked cross-cultural validity, as different factor structures emerged with different languages (i.e., French, English, Dutch, Spanish, and German) and cultures (i.e., Canadian, American, Dutch, Spanish, and German). Furthermore, Norton [32] investigated the psychometric properties and the factor structure of the IUS in four different racial groups (African, Caucasian, Hispanic, and Southeast Asian) and reported differences in the factor structure across groups, despite substantially observing similar reliability and validity values. To note, most of these studies were based on under- or post-graduate students; factor analyses were carried out on adult community samples only in a few cases [27, 28]. A further issue concerning the validity of the IUS was the “GAD-specific” nature of some of its items (e.g. “My mind can’t be relaxed if I don’t know what will happen tomorrow”), which raised further questions about its capability to capture the core IU construct [3, 33] in a trans-diagnostic context. In the attempt to overcome these issues, the original IUS was reduced to a 12-item scale by Carleton et al. [26].

### The Intolerance of Uncertainty Scale-12

Following the previous considerations, Carleton et al. [26] developed the IUS-12, a 12 item-questionnaire consisting of two factors they had identified in a subset of the items in the original measure. The first, Prospective IU, expresses the propensity of individuals toward active information seeking as a way to reduce uncertainty/increase certainty. The second, Inhibitory IU, refers to avoidance-oriented responses to uncertainty, i.e. an inhibition of actions or experience which is caused by uncertainty [24, 26, 34]. The authors recommended the use of the IUS-12 total score over the IUS to evaluate general IU as the latter was still too close to a measure of worry [26]. The IUS-12’s correlated two-factor structure was initially tested in two large undergraduate samples [26] and further replicated in several studies employing also community [3] and clinical [34–37] samples. Its cross-cultural validity (i.e., White and Black university students as well as Dutch undergraduate students) was also demonstrated [38, 39]. Good internal consistency of the IUS-12 total score and of the two subscales has been observed in non-clinical samples [3, 36, 39, 40], as well as in samples of people diagnosed with GAD [36], OCD [35], and comorbid anxiety and depression [37]. Furthermore, the IUS-12 has shown medium-high correlations with measures of worry, anxiety, depression, social anxiety, and general distress, and a stronger correlation with the Perfectionism/certainty scale of the Obsessive Beliefs Questionnaire-44 (OBQ-44) [26, 35–37, 39–41]. Taken together, the previous findings on the convergent and divergent validity of the IUS-12 support the trans-diagnostic nature of IU.

More recently, Hale et al. [31] pointed out that Carleton et al. [26] did not provide empirical justification for deriving a total score on the IUS-12. Therefore, they analyzed the factor structure of the IUS-12 in a sample of American psychology undergraduates and showed that a bifactor model fitted the data better than the unidimensional model and Carleton et al.’s correlated two-factor solution. They obtained evidence of a strong general factor and of weak and unreliable Prospective and Inhibitory group factors. Similarly, Lauriola, Mosca, and Carleton [42] tested the factor structure of an Italian translation of the IUS-12 in a sample of psychology university students and also observed that a bifactor model fitted the sample data better than the alternative models. Finally, Shihata, McEvoy and Mullan [43] recently reported that a bifactor model provided the best fit in Australian psychology undergraduate and clinical samples. In both these studies, similar findings to Hale et al. [31] were found, namely good reliability of the general factor and lower reliability for the group factors.

It should be stressed, however, that a better fit of the bifactor model might not necessarily imply that the factor structure of the IUS-12 follows a bifactor structure rather than a unidimensional or a correlated two-factor solution. Indeed, both the substantive interpretation of the bifactor solution as the true factor model underlying specific cognitive or psychological phenomena, and the fact that the bifactor might be advantaged in terms of fit indexes over other models, are still under debate [44–47]. Notwithstanding these issues, the bifactor model can be used to assess whether the group factors can be considered independent and auto-sufficient scales or alternatively whether the unit-weighted total score can be used as a univocal measure of a unidimensional construct without introducing excessive bias due to any multi-dimensionality [48–51]. In the present manuscript the bifactor model is thus considered and investigated more for the latter reasons rather than as a viable alternative model to the unidimensional or the correlated two-factor solution.

### The current study

Different adaptations of the same questionnaire for children or adults often lead to different items and factor structures, thus making the interpretation and comparison of scores difficult [52]. In order to create a version of the IUS-12 which is suitable to people of different ages, Walker, Birrell, Rodgers, Leekam, and Freeston [53] rephrased some of the items of the IUS-12 and thus developed the IUS-Revised (IUS-R), a measure assessing IU across the lifespan. In particular, the language was simplified so that it can be easily read by an average 11-year-old student (Flesch Reading Ease = 81.5) [53]. To date, the IUS-R has proven to be a reliable and valid measure of IU in different samples: non-clinical samples of British and Spanish young people aged 6–8 years, 9–11 years, 12–14 years, and undergraduates [54]; healthy young people aged 11–17 [55]; children and adolescents with diagnoses of Autism or Asperger’s syndrome [56, 57]. Preliminary data supporting the adequacy of the IUS-R have been reported in a sample of Italian undergraduate students: Bottesi et al. [58] showed that Carleton et al.’s correlated two-factor model [26] fitted the data better than a single-factor model. Moreover, the Italian IUS-R showed acceptable internal consistency (total score: *α* = .80; Prospective IU: *α* = .68; Inhibitory IU: *α* = .79) and adequate construct validity. Furthermore, the IUS-R total score significantly predicted worry, even after controlling for somatic anxiety and depressive symptoms. However, Bottesi et al. [58] did not address some important issues, namely, the factor structure of the questionnaire in a community sample, an investigation of a bifactor model, gender and age invariance, and psychometric properties such as temporal stability.

The present study aims to fill some gaps in the extant literature on the assessment of IU as measured by means of the IUS, the IUS-12, or the IUS-R in a detailed psychometric examination of the Italian IUS-R. In our opinion, expanding knowledge about the measurement of IU through the IUS is highly desirable and warranted: providing clinical researchers and practitioners with a measure capable of adequately capturing the IU construct during screening and/or assessment phases has indeed clear implications in terms of both clinical evaluation and treatment planning.

First, we examine the factor structure of the IUS-R in a large community sample given that a significant proportion of studies on the various IUS measures have been conducted among psychology undergraduates [2, 17, 26, 31, 42, 43, 58] preventing generalization to community samples. Second, we address measurement invariance across gender and age. To our knowledge, to date only one study assessed gender invariance among university students [31], whereas none have examined age invariance. Third, we address one-month temporal stability and longitudinal measurement invariance in a sample of undergraduates: to our knowledge, only Khawaja and Yu [36] provided information about test-retest reliability (two-week interval) of the IUS-12 in a similar group (*r* = . 77), but they did not test measurement invariance over time. Finally, we address the reliability of the Prospective and Inhibitory subscales, which have been previously suggested to be unreliable [31, 43]. As the IUS-R is adapted from the IUS-12, most of the expected results were based on previous literature on the original version of the questionnaire. Specifically:

1. The bifactor model was expected to show a superior fit when assessing the structure of the Italian IUS-R in a large community sample. Consistent with recent evidence [31, 42, 43], although the correlated two-factor model was expected to show better fit than the unidimensional model, the bifactor model was expected to support a unidimensional structure and high reliability of the Italian IUS-R unit-weighted total score (please note that, for readability reasons, from this point on we will simply define it as the “IUS-R total score”). In addition, although the Prospective and Inhibitory subscales were not expected to be reliable, item loadings on the group factors were expected to be stronger than in the IUS-12 as a result of the modifications of the items carried in the IUS-R version.
2. The correlations between the Italian IUS-R scores and demographic variables such as age and education were expected to be small (e.g., *r* < |.10|).
3. The Italian IUS-R was expected to possess good convergent validity. Based on previous literature on the IUS-12 and the preliminary data reported by Bottesi et al. [58], we anticipated to observe medium-to-large positive correlations (e.g., *r ≥* .30 to < .50) with measures of negative problem orientation (NPO), worry, anxiety, depression, general distress, OC symptoms, and a strong association with a concurrent measure of IU [35].
4. In line with Hale et al. [31], no significant bias in the items was expected across genders. As only a few studies have reported on gender differences [39, 58], and in line with preliminary findings by Bottesi et al. [58], no gender differences were expected. In particular, measurement invariance across gender was expected. Similarly, as the IUS-R was developed to assess IU across lifespan, measurement invariance was also expected across different age groups.
5. Given that the original IUS and its derivatives are conceptualized as measuring a dispositional feature and in light of previous literature, temporal stability of the IUS-R was expected to be strong and invariant in an undergraduate sample [36].

## Materials and methods

### Participants and procedure

A community sample made up of 761 people (302 men and 459 women), living in different midsized communities in northern, central, and southern Italy, were recruited through advertisements requesting volunteers for psychological studies. Their mean age was 35.86 (*SD* = 14.01, *range* = [17, 78]) and their mean years of education were 14.36 (*SD* = 3.73, *range* = [5, 30]). Marital status was 54.7% single/living alone, 39.3% married/in a domestic relationship, 5.1% separated/divorced, and 0.9% widowed. The employment profile of the sample was: 43.8% full-time employment, 28.1% student, 7.9% part-time employment, the 3.8% full-time homemaker, 3.9% occasionally employed, 3.3% retired, 3.4% unemployed, 0.4% unable to work due to disability, and 5.4% other. Exclusion criteria were current psychological disorders, major neurocognitive disorder, and intellectual disability (intellectual developmental disorder). Data from this sample was used to assess the factor structure, reliability, measurement invariance, and convergent validity. Data were collected between 2012 and 2017 in four different but related studies, each employing slightly different batteries of self-report measures. The resulting datasets were merged to obtain the final community sample of this study. Therefore, not all individuals completed all the same self-report questionnaire measures, but all individuals completed the IUS-R.

A further non-clinical sample of 163 undergraduate students (47 males and 116 females) attending the second year of university at the School of Psychology was recruited to test one-month temporal stability and longitudinal measurement invariance. Exclusion criteria were the same as for the community sample. Their mean age was 21.16 (*SD* = 2.64, *range* = [19, 48]), and mean years of education were 13.22 (*SD* = .79, *range* = [12, 18]); 46.6% were single/living alone and 53.4% were in a relationship.

All participants were informed of the study’s aims and gave their written, informed consent before entering the study; no incentives were offered. Participants filled in a socio-demographic form and a battery of self-report questionnaires. The research was conducted in accordance with the Declaration of Helsinki and was approved by the Ethics Committee of Psychological Sciences of the University of Padova. This research did not receive any specific grant from funding agencies in the public, commercial, or not-for-profit sectors.

### Measures

In the present subsection all the measures considered in the manuscript are listed together with their values of Cronbach’s alpha and the associated confidence intervals and sample sizes. It should be noted, however, that Cronbach’s alpha has been here reported mostly for comparative reasons with the extant literature. Readers should exercise caution in interpreting Cronbach’s alpha, as the reported measures largely differ both in number and homogeneity of the items, so that any interpretation should be driven by considerations on the context and the scope and length of the measure [59]. A more methodologically sound comparison between the measures should be based on cumulative reliability functions obtained by means of re-sampling techniques [60]. In the present study, examination of the reliability of the IUS-R scales and its Prospective and Inhibitory subscales will be addressed by means of omega composite reliability coefficients associated with the bifactor solution. As a final note, Table 1 lists published means and standard deviations of the Italian version of the measures, together with their estimated values in the current community sample.

**Table 1:** Means and standard deviations for all measures (Italian versions) from published studies (population parameters) and the current sample.

	Population			Sample estimates of population parameters			
	Mean	SD	Items	Mean	SD	Skew.	Kurt.
OBQ-87-T	45	11.2	13	37.93	14.28	.24	-.52
NPOQ	24.35	9.60	12	24.41	9.60	.86	.31
PSWQ	48.8	13.8	16	42.88	12.11	.38	-.17
BAI	11	8.73	21	9.38	8.68	1.73	4.20
BDI-II	8.2	5.6	21	6.37	5.40	1.72	6.92
DASS-21	12.3	8.3	21	14.16	7.39	.20	-.42
OCI-R	7.8	7.6	18	10.80	7.47	1.12	1.71
SPS	11.88	8.30	20	15.55	8.93	.57	-.40

OBQ-87-T = Obsessive Beliefs Questionnaire-87 tolerance of uncertainty subscale; NPOQ = Negative Problem Orientation Questionnaire; PSWQ = Penn State Worry Questionnaire; BAI = Beck Anxiety Inventory; BDI-II = Beck Depression Inventory–Second Edition; DASS-21 = Depression Anxiety Stress Scale-21; OCI-R = Obsessive Compulsive Inventory—Revised; SPS = Social Phobia Scale.

*The Intolerance of Uncertainty Scale-Revised* (IUS-R; Italian version by Bottesi et al. [58]; the Italian version of the IUS-R can be found in the S1 File) consists of 12 items assessing IU. Respondents are asked to rate the extent to which each statement applies to themselves on a 5-point Likert scale; as previously outlined, promising preliminarily psychometric properties have been reported [58]. Almost all participants (758 community individuals and 163 undergraduate students) completed the IUS-R. In the community sample, Cronbach’s alpha was *α* = .87 (*CI* = [.85, .88], *N* = 758). The IUS-R contains two subscales, *Prospective IU* (IUS-R-P), and *Inhibitory IU* one (IUS-R-I). Cronbach’s alphas for the subscales were respectively *α* = .78 (*CI* = [.76, .81], *N* = 758) and *α* = .86 (*CI* = [.84, .87], *N* = 758).

*The Obsessive Beliefs Questionnaire-87* (OBQ-87; Italian version by Sica et al. [61]) consists of 87 items assessing the dysfunctional belief domains believed to be involved in the onset and maintenance of OCD. The Italian version of the measure has demonstrated good internal consistency and test-retest reliability. Given the purpose of the present study, only the subscale measuring tolerance of uncertainty (OBQ-87-T) was used to assess convergent validity with the IUS-R. Cronbach’s alpha in the community sample was *α* = .89 (*CI* = [.88, .91], *N* = 213).

*The Negative Problem Orientation Questionnaire* (NPOQ; Italian version by Bottesi & Ghisi [62]) consists of 12 items assessing the individual’s approach to problems, including beliefs that problems are threatening, low self-confidence about abilities to solve problems, and pessimism about problem resolution (i.e., NPO). The Italian version of the NPOQ has shown adequate internal consistency, test-retest reliability, convergent, and discriminant validity. Cronbach’s alpha in the community sample was *α* = .93 (*CI* = [.92, .94], *N* = 429).

*The Penn State Worry Questionnaire* (PSWQ; Italian version by Morani, Pricci, & Sanavio [63]) consists of 16 items measuring the tendency to worry excessively and uncontrollably. Psychometric properties of the Italian version were adequate. In the community sample, Cronbach’s alpha was *α* = .91 (*CI* = [.89, .92], *N* = 429).

*The Beck Anxiety Inventory* (BAI; Italian version by Sica, Coradeschi, Ghisi, and Sanavio [64]) consists of 21 items assessing the severity of anxiety over the previous week. The Italian version of the BAI showed excellent internal consistency and good test-retest reliability; Cronbach’s alpha in the community sample was *α* = .90 (*CI* = [.89, .91], *N* = 256).

*The Beck Depression Inventory-II* (BDI-II; Italian version by Ghisi, Flebus, Montano, Sanavio, & Sica [65]) consists of 21 items evaluating the severity of depression over the previous two weeks. The BDI-II showed high internal consistency and good test-retest reliability in the Italian version. In the community sample, Cronbach’s alpha was *α* = .83 (*CI* = [.81, .85], *N* = 258).

*The Depression Anxiety Stress Scales -21* (DASS-21; Italian version by Bottesi et al. [66]) consists of 21 items assessing depression, anxiety, and stress over the previous week. Findings on the Italian version suggested that the use of the total score, measuring a general distress factor, might be more appropriate than scoring the three subscales separately; the total score of the Italian version showed excellent internal consistency [66]. In the community sample, Cronbach’s alpha was *α* = .90 (*CI* = [.88, .91], *N* = 170).

*The Obsessive Compulsive Inventory-Revised* (OCI-R; Italian version by Sica et al. [67]) consists of 18 items assessing the distress caused by several OC symptoms in the past month; internal consistency in the Italian version was good. Given the purpose of the present study, the OCI-R total score was used. The Cronbach alpha value for the total score observed in the community sample was *α* = .84 (*CI* = [.82, .86], *N* = 127).

*The Social Phobia Scale* (SPS; Italian version by Sica et al. [68]) consists of 20 items assessing situations that involve being observed by other. The Italian version proved to be highly reliable and stable. Cronbach’s alpha in the community sample was *α* = .87 (*CI* = [.85, .89], *N* = 127).

### Data analysis

Statistical analyses were performed with R [69] and the packages *lavaan* [70], *semTools* [71], and *psych* [72]. Confirmatory factor analyses (CFAs) were carried with the WLSMV robust estimator for ordinal data to test a unidimensional model, a correlated two-factor model, and a standard bifactor model accounting for both Prospective IU and Inhibitory IU group factors together with a general IU factor. As the bifactor model might be advantaged by traditional fit indexes in comparison to more parsimonious models [44–47], though it might not necessarily represent the true model behind the IU construct, significance of the fit indexes was not considered as the main criterion for model selection. Rather, considerations on model selection were driven by the results provided by the standard bifactor model as a tool to investigate the dimensionality of the test [48–51]. In addition, a modified version of the standard bifactor model was considered, in which the correlation between the group factors is freed (rather than constrained to be orthogonal under the standard model), in order to shed more light on the effects of a common factor in a correlated two-factor model. Considerations as to the factor structure of the IUS-R were then drawn based on a comparative discussion of these models. As to fit indexes, models were compared by means of chi-squared difference tests [73, 74] and a *ΔCFI* criterion (|*ΔCFI*| of < .01 suggests no significant difference) [75]. Fit indexes and tests were evaluated by the following criteria: *χ*^*2*^ should not be significant (although its dependence on sample size is largely acknowledged); Normed Chi square *χ*^*2*^/df < 2.0; Comparative Fit Index (*CFI*) and Tucker Lewis Index (*TLI*) > .95; Root Mean Square Error of Approximation (*RMSEA*) < .05; Standardized Root Mean square Residuals (*SRMR*) < .08; Weighted Root Mean square Residuals (*WRMR*) < 1.0 [76].

Similarly, considerations on the reliability of the IUS-R and its subscales IUS-R-P and IUS-R-I were given in terms of composite reliability rather than Cronbach’s *α*. In presence of broad (and possibly multidimensional) constructs with congeneric items, Cronbach’s alpha can underestimate reliability and is neither a measure of dimensionality nor of internal consistency [77]. For this reason, composite reliability coefficients have been developed to account for the different loadings of the items. In the present work, composite reliability was assessed by means of Raykov’s *ρ* [78], Bentler’s *ω* [79], and McDonald’s *ω*_*T*_ and *ω*_*h*_ [80]. Typically, values > .70 were considered evidence of good reliability. The first two coefficients can be seen as a ratio between the reliable variance and the total test variance. The main difference between Raykov’s *ρ* and Bentler’s *ω* is that the former accounts for uncorrelated measurement errors while the latter considers correlated errors. Both coefficients are based on the model implied covariance matrix to assess the total test variance. In contrast, Mc Donald’s *ω*_*h*_ is based on the observed covariance matrix. The main difference between *ω*_*T*_ and *ω*_*h*_, is that the former builds the reliable variance based on the loadings of all the factors while the latter only on the loadings of the general factor of the bifactor model. Of particular interest, *ω*_*h*_ is unaffected by the number of items in the test.

In addition, Explained Common Variance (*ECV* > .60) was computed as the percent of common variance due to the general factor and as an indicator of unidimensionality [49]. In relation to *ECV* and *ω*_*h*_, the Percentage of Uncontaminated Correlations (*PUC*) was also controlled to verify whether any structural bias induced by forcing a multidimensional structure into a unidimensional scale could be disregarded so that practitioners are able to use the total score of the questionnaire as a univocal reliable measure of the common factor [51]. Generally, if PUC is higher than .80 then the other indexes are less relevant, otherwise they all need to be considered [51]. The Average Variance Extracted (*AVE*) was provided to compare variances due to construct and measurement error. Generally, values >.5 are considered acceptable and values >.7 are considered very good [81].

In the community sample, Pearson correlations were then calculated between the IUS-R total score with age and years of education; eta squared (*η*^*2*^) was used to quantify the magnitude of the effect. Following Cohen’s [82] criteria, .01 is considered small, .06 medium and .14 large. Correlations of the IUS-R total score and subscores with the other measures in the community sample were also computed to assess convergent validity. Partial correlations were calculated, controlling for the effect of the other constructs. Correlations were also calculated between all the measures and the predicted factor scores for the latent dimensions of the bifactor model in the community sample order to look for some evidence that the general common factor is not simply a method factor. Values of |.30| and |.50| were respectively considered moderate and large.

In addition, since the IUS-R was developed to be suitable across the lifespan and is also supposed to be gender invariant, Measurement Invariance (MI) with respect to gender and age was independently assessed using Multi-Group Confirmatory Factor Analysis (MGCFA) by inspecting the fit of 1) separate bifactor models for the groups (men and women, or different age groups); 2) configural invariance (loading and threshold parameters differ across groups); 3) metric invariance (only thresholds differ across groups); and 4) scalar invariance (loadings and thresholds cannot differ across groups). Since WLSMV was used, when testing scalar invariance a difference test for both factor loadings and thresholds simultaneously was applied, thus directly comparing configural and scalar models, as suggested by Muthén and Muthén [83].

Finally, temporal stability and reliability of the IUS-R in the undergraduate sample were assessed using Pearson’s correlations, Intra-Class Correlations (*ICC*), and longitudinal MI [84]. Bifactor models were fitted separately in the test and retest conditions, then configural, metric, and scalar invariance were assessed by fitting separate bifactor models to the two conditions, correlating all factors and all item residuals to account for their longitudinal dependence. MI between community and undergraduate samples was examined to test whether the results of the longitudinal MI on the undergraduate sample could be extended to the community sample.

## Results

### Descriptive statistics

All IUS-R items were approximately symmetric (|skewness|≤1), except for items 9, 11, and 12 that were skewed (|skewness|>1). Eight items showed negative excess kurtosis between 0 and -1, except item 3 (-1.05), and items 9, 11 and 12 which showed positive kurtosis (.65, 1.13, and 2.38 respectively). Mardia’s multivariate test indicated significant multivariate skewness and kurtosis.

### Factor structure and reliability

As analyses were conducted on complete data, three participants were removed from the community sample (N = 758). Table 2 reports the fit indexes for a) the unidimensional model, b) the correlated two-factor model, c) the standard bifactor model, and d) a bifactor model in which the correlation between the group factors was freed, which is essentially model b) with the addition of a common factor. As can be seen in Table 2, the bifactor model c) showed the strongest fit indexes as expected when compared to the unidimensional and correlated two-factor models. Note that model d) could be considered the best fit, but this model was used only to investigate the relationships between the other models as will be discussed later. The unidimensional model showed the poorest robust fit indexes while all loadings were significantly different from zero and in the range [.371, .819], with robust standard errors in the range [.017, .031]. The correlated two-factor model showed moderate robust fit indexes; again, all loadings were also significantly different from zero and in the range [.404, .849], with robust standard errors in the range [.016, .032]. The estimated correlation between the Prospective IU and Inhibitory IU group factors in the bifactor model was .75 (*SE* = .022; *z* = 33.885, *p* < .001), which is slightly lower than the correlation between the predicted factor scores for the Prospective IU and Inhibitory IU group factors (*r* = .83, *CI* = [.808, .852], *t*(758) = 41.194, *p* < .001). Interestingly, both these correlations are higher than the observed correlation between the IUS-R-P and IUS-R-I subscale scores (*r* = .59, *CI* = [.545, .637], *t*(758) = 20.254, *p* < .001). This suggests a strong correlation between the latent factors in the correlated two-factor model, which possibly hints to the existence of a second order factor or of a general common factor as in the bifactor model. The standard bifactor model showed good robust fit indexes. As expected, results from the scaled *χ*^*2*^ difference test and the *ΔCFI* criterion indicated that the bifactor solution fitted the data significantly better than both the unidimensional and correlated two-factor models. Again, it is important to stress that the superior fit indexes of the bifactor model does not imply that IU is best described by such a model. Rather, the bifactor model can be used as a tool to examine the dimensionality and reliability of the scales.

**Table 2:** Fit statistics for the unidimensional, correlated two-factor, and bifactor models. Scaled difference Δχ^2^ test and ΔCFI criterion are also reported.

Model	*N*	*χ*^*2*^	*df*	*χ*^*2*^*/df*	*p*	*CFI*	*TLI*	*RMSEA*	*SRMR*	*WRMR*
a) Unidim.	758	729.704	54	13.513	< .001	.920	.903	.129	.082	2.139
b) Correlated two-factors	758	327.858	53	6.186	< .001	.968	.960	.083	.054	1.373
c) Bifactor	758	137.670	42	3.278	< .001	.989	.982	.055	.032	.781
d) Bifactor with correlated traits	758	102.575	41	2.502	< .001	.993	.988	.045	.024	.658
Comparison	*N*	*Δχ*^*2*^	*df*	*χ*^*2*^*/df*	*p*	*ΔCFI*				
c) vs a)	758	315.120	12	26.260	< .001	.068				
c) vs b)	758	116.020	11	10.547	< .001	.021				
d) vs c)	758	15.163	1	15.163	< .001	.004				

CFI = Comparative Fit Index; TLI = Tucker Lewis Index; RMSEA = Root Mean Square Error of Approximation; SRMR = Standardized Root Mean square Residuals; WRMR = Weighted Root Mean square Residuals.

As can be seen in Table 3, the loadings on the general factor of the bifactor model were all well-estimated and in the .5 to .8 range, except for items 3 and 4 with values of .345 and .466 respectively which are still considered acceptable. Interestingly, although the loadings were slightly lower than those in previous reports [31, 43], their values were consistent (including the lower values for items 3 and 4) with those from the previous Italian sample [42]. Items 1 and 2 on the Prospective IU group factor were problematic as their loadings on the group factor had opposite signs to all other items on the same group factor. To note, when the correlation between the Prospective IU and Inhibitory IU group factors in the bifactor model was freed, most loadings of the general common factor decreased in magnitude, all loadings of the Inhibitory IU factor increased in magnitude, whereas almost all loadings of the Prospective IU group factor became negative (although some of them were not significantly different than zero). In addition, the correlation between the latent group factors was estimated as -.66 (*SE* = .066, *z* = -9.910, *p* < .001), which is of the same magnitude, but of the opposite sign than the correlation estimated in the correlated two-factor solution. This result suggests that, although both Prospective and Inhibitory group factors contribute in describing the IU construct, their specific group content might actually be of an opposite nature. For further details together with further discussion of the loadings in the present and previous studies [31, 42, 43], please see the discussion section.

**Table 3:** Standardized loadings for the standard bifactor model and the bifactor with correlated group factors. For each loading, the associated robust standard error in round brackets, together with z- value and significance, are provided.

	Standard bifactor			Bifactor with correlated group factors		
Items	General	Prospect.	Inhibitory	General	Prospect.	Inhibitory
1—When things happen suddenly, I get very upset	.746(.025) 29.735***	-.182(.059) -3.099**		.378(.115) 3.298**	-.673(.073) -9.276***	
2—It bothers me when there are things I don’t know	.677(.028), 24.407***	-.166(.056) -2.941**		.359(.103) 3.472**	-.590(.074) -8.026***	
3—People should always think about what will happen next. This will stop bad things from happening	.345(.039) 8.924***	.378(.053) 7.110***		.528(.035) 14.885***	.032(.095) .335	
4—Even if you plan things really well, one little thing can ruin it	.466(.033) 14.120***	.147(.054) 2.719**		.427(.056) 7.648***	-.237(.087) -2.722**	
5—I always want to know what will happen to me in the future	.612(.031) 19.533***	.362(.050) 7.174***		.690(.041) 16.884***	-.185(.117) -1.588	
6—I can’t stand it when things happen suddenly	.780(.021) 37.404***	.157(.050) 3.134**		.656(.079) 8.285***	-.452(.113) -3.999***	
7—I should always be prepared before things happen	.698(.032) 22.143***	.491(.048) 10.233***		.826(.037) 22.094***	-.174(.136) -1.284	
8—Feeling unsure stops me from doing most things	.615(.026) 23.359***		.533(.030) 17.578***	.427(.082) 5.216***		.690(.053) 12.968***
9—When I’m not sure what to do I freeze	.593(.031) 18.977***		.666(.030) 20.161***	.348(.100) 3.487***		.815(.045) 18.272***
10—When I don’t know what will happen, I can’t do things very well	.661(.025) 26.865***		.436(.029) 15.024***	.500(.072) 6.914***		.620(.059) 10.498***
11—The smallest concern can stop me from doing things	.580(.031) 18.564***		.580(.034) 17.061***	.324(.097) 3.352***		.771(.043) 17.792***
12—I must get away from all things I am unsure of	.633(.031) 20.373***		.392(.039) 10.094***	.498(.070) 7.144***		.560(.062) 9.052***

Significance levels:

*** *p* < .001,

** *p* < .01.

Reliability indexes for the IUS-R general factor score as well as for the Prospective IU and Inhibitory IU group factors in the bifactor model, together with their respective correlations with the total score (unit-weighted scores), are reported in Table 4. As measures of unidimensionality we calculated the Percentage of Uncontaminated Correlations (*PUC* = .53, being lower than .80 is also necessary to control for the other indices), the Explained Common Variance (ECV = .70, good), and the Average Variance Extracted (AVE = .563, acceptable).

**Table 4:** Reliability of the Italian IUS-R (community sample). Upper panel: Composite reliability coefficients for the general factor and group factors from the standard bifactor model and for the total score. Lower panel: Cronbach’s alpha for unrefined score and correlations with factor score.

	Bifactor model			Total
	General	Prospective IU	Inhibitory IU	
Composite reliability coefficients				
Raykov’s *ρ*	.723	.174	.458	.896
Bentler’s *ω*	.778	.066	.349	.896
McDonald’s *ω*_*h*_	.781	.066	.352	.899
Unrefined factor scores				
Cronbach’s α*	.899	.819	.901	.899
Correlations with associated refined factor scores	.948	.360	.729	-

*Cronbach’s *α* values for the unit-weighted summed scores have been derived from polychoric correlations; thus their values slightly differ from the standard calculations reported in the Measures section.

All estimates of composite reliability point towards a unidimensional scale and a reliable total score (*mean IUS-R* = 26.73, *SD* = 8.20). Together, *ω*_*T*_ and *ω*_*h*_ indicated that almost 87% of the reliable variance can be attributed to the general factor (obtained as the ratio between the omega for the general factor .781 and the omega for the total score .899 in Table 4) [50]. For comparison, the composite reliability calculated for the unidimensional model is Bentler’s *ω* = .88, and *ω*_*h*_ = .91. In addition, both group factors may be unreliable, although the reliability for the Inhibitory IU group factor was higher than for the Prospective IU group factor. Cronbach’s *α* for the total scores based on the group factors might thus be inflated by the contribution of the general factor. While the general factor score shows a strong correlation with the associated IUS-R total score (see Table 4), the same does not hold for the group factors and the unrefined factor scores. Taken together these results indicate relative unidimensionality despite some multidimensionality and that the total score is a good enough indicator of the general factor; please see the next section and the discussion section for further comments on the subscales.

### Associations with demographic variables and convergent validity of the IUS-R and of the predicted factor scores

Given that the previous analyses indicated acceptable unidimensionality (despite some underlying multidimensionality), correlations were examined for the IUS-R scale and its Prospective IU and Inhibitory IU subscales (see Table 5, upper panel). The same analyses were conducted for the predicted factor scores from the bifactor model (see Table 5, lower panel). The latter simultaneously aimed to assess whether the latent common factor correctly represented the IU construct rather than a method factor, and to show to what extent predicted Prospective IU and Inhibitory IU factor scores behaved like independent and reliable group factors. It should be noted that pairwise deletion of missing data was applied to the combined data set since, as previously mentioned, the community sample was obtained by merging four different studies with different batteries of measures. Readers should also be careful in interpreting the correlations for the same reason caution was advised in interpreting Cronbach’s alpha in the Measures section.

**Table 5:** Inter-correlations between the measures and the IUS-R scores (upper panel) or the IUS-R predicted factor scores (lower panel). Sample size for each correlation is reported within round brackets. Non-available correlations are reported as NA.

	**Intercorrelations of the total scores with the other external measures**									
	**IUS-R-P**	**IUS-R-I**	**OBQ-87-T**	**NPOQ**	**PSWQ**	**BAI**	**BDI-II**	**DASS-21**	**OCI-R**	**SPS**
**IUS-R**	.92*** (758)	.86*** (758)	.56*** (213)	.63*** (424)	.53*** (425)	.38*** (256)	.30*** (258)	.25*** (160)	.42*** (127)	.50*** (127)
**IUS-R-P**		.59*** (758)	.56*** (213)	.56*** (424)	.49*** (426)	.33*** (256)	.26*** (258)	.18*** (161)	.37*** (127)	.45*** (127)
**IUS-R-I**			.45*** (213)	.57*** (425)	.45*** (425)	.34*** (256)	.27*** (258)	.28*** (161)	.41*** (127)	.45*** (127)
**OBQ-87-T**				.56*** (213)	.55*** (213)	.42*** (83)	.54*** (85)	.22*** (127)	.52*** (127)	.36*** (127)
**NPOQ**					.60*** (425)	.44*** (256)	.42*** (258)	.35*** (161)	.37*** (127)	.39*** (127)
**PSWQ**						.34*** (256)	.41*** (258)	.36*** (161)	.28*** (127)	.38*** (127)
**BAI**							.23*** (255)	NA	NA	NA
**BDI-II**								NA	NA	NA
**DASS-21**									.30*** (127)	.10 (127)
**OCI-R**										.25* (127)
	**Intercorrelations of the predicted factor scores with the other external measures**									
	**IUSP**	**IUSI**	**OBQ-87-T**	**NPOQ**	**PSWQ**	**BAI**	**BDI-II**	**DASS-21**	**OCI-R**	**SPS**
**GEN**	.14*** (758)	.13* (758)	.57*** (213)	.60*** (424)	.55*** (425)	.36*** (256)	.30*** (258)	.25* (160)	.41*** (127)	.49*** (127)
**IUSP**		-.17*** (758)	.17 (213)	.09 (424)	-.01 (425)	.07 (256)	.03 (258)	-.16 (160)	.10 (127)	.07 (127)
**IUSI**			.11 (213)	.26*** (424)	.15 (425)	.18 (256)	.17 (258)	.18 (160)	.14 (127)	.18 (127)

IUS-R = The Intolerance of Uncertainty Scale-Revised scale; IUS-R-P = Prospective subscale of IUS-R; IUS-R-I = Inhibitory subscale of the IUS-R; GEN = Predicted values of the general factor of the bifactor model; IUSP = predicted values for the Prospective factor, IUSI = predicted values for the Inhibitory factor; OBQ-87-T = Obsessive Beliefs Questionnaire-87 tolerance of uncertainty subscale; NPOQ = Negative Problem Orientation Questionnaire; PSWQ = Penn State Worry Questionnaire; BAI = Beck Anxiety Inventory; BDI-II = Beck Depression Inventory—Second Edition; DASS-21 = Depression Anxiety Stress Scale-21; OCI-R = Obsessive Compulsive Inventory—Revised; SPS = Social Phobia Scale. Significance levels:

* < .05,

** < .01,

*** < .001.

For the demographic variables, Pearson’s product-moment correlations of the IUS-R total score with age was significantly different than zero but shows a small effect (*r* = -.14, *CI* = [-.210, -.070], *t*(759) = -3.899, *p* < .001, *η*^*2*^ = .022). In the next section MI with respect to age is examined formally. Correlation with education was non-significant (*r* = -.002, *CI* = [-.070, .070], t(759) = -.060, *p* = .95). There was also no significant difference in the total score between genders (*t*(693.82) = -.28, *p* = .78, *η*^*2*^ = .0001). Correlations of the IUS-R and its subscales IUS-R-P and IUS-R-I with the other study measures in the community sample are reported in Table 5 (upper panel). As can be seen in the top row, the strongest correlation with external measures was found between the IUS-R and the NPOQ, immediately followed by medium correlations between the IUS-R and the OBQ-87 tolerance of uncertainty, PSWQ, SPS, and OCI-R, whereas correlations with BAI, BDI-II, and DASS-21 were weak.

Next, partial correlations were conducted on the largest possible subsample (*N* = 213) that simultaneously provided complete data for the OBQ-87 tolerance of uncertainty subscale, NPOQ, and PSWQ, i.e. the three measures most strongly correlated with the IUS-R. The partial correlations between the IUS-R and both the OBQ-87 tolerance of uncertainty (*r* = .26) and the NPOQ (*r* = .47) decreased in strength but remained significant (*p* < .001) after controlling for the other measures; the correlation between the IUS-R and PSWQ was no longer significant (*r* = .09).

Moving now to the factor scores, Table 5 (lower panel) reports the correlations of the predicted factor scores for the standard bifactor. As it can be seen, the predicted factor score of the general common factor (GEN) showed essentially the same strength of correlations with the other measures as the total score in Table 5 (upper panel) (which might be expected as from Table 4 their correlation is about .95). However, the predicted values of the factor scores for the Prospective IU (IUSP) and the Inhibitory IU (IUSI) group factors did not show convergent validity with the other external measures. This appears to support the essential unidimensionality of the construct as well as the unreliability of the subscales.

### Measurement invariance of the bifactor model

Steps to verify the MI of the bifactor model in the community sample are summarized in Table 6 for the variable gender and in Table 7 for the (categorized) variable age.

**Table 6:** Fit statistics for the bifactor model tested for invariance across gender (community sample).

Model	*N*	*χ*^*2*^	*df*	*χ*^*2*^*/df*	*p*	*CFI*	*TLI*	*RMSEA*	*SRMR*	*WRMR*
Men	301	82.136	42	1.956	< .001	.985	.977	.056	.044	.645
Women	457	71.262	42	1.697	.003	.995	.992	.039	.026	.550
a) Configural inv.	758	154.116	84	1.835	< .001	.992	.988	.047	.033	.848
b) Metric inv.	758	239.614	108	2.219	< .001	.985	.982	.057	.057	1.470
c) Scalar inv.	758	288.360	140	2.060	< .001	.983	.984	.053	.045	1.431
Comparison	*N*	*Δχ*^*2*^	*df*	*χ*^*2*^*/df*	*p*	*ΔCFI*				
b) vs a)	758	48.181	24	2.010	.002	.007				
c) vs a)	758	107.360	56	1.917	< .001	.009				

CFI = Comparative Fit Index; TLI = Tucker Lewis Index; RMSEA = Root Mean Square Error of Approximation; SRMR = Standardized Root Mean square Residuals; WRMR = Weighted Root Mean square Residuals.

**Table 7:** Fit statistics for the bifactor model tested for invariance across age groups (community sample).

Model	*N*	*χ*^*2*^	*df*	*χ*^*2*^*/df*	*p*	*CFI*	*TLI*	*RMSEA*	*SRMR*	*WRMR*
18–25	324	82.879	42	1.973	< .001	.988	.981	.055	.037	.598
26–45	215	83.357	42	1.985	< .001	.983	.974	.068	.053	.646
>46	219	62.199	42	1.481	< .001	.992	.987	.047	.040	.536
a) Configural inv.	758	227.698	126	1.807	< .001	.988	.981	.057	.042	1.031
b) Metric inv.	758	223.499	174	1.284	< .001	.994	.993	.034	.056	1.417
c) Scalar inv.	758	381.856	236	1.618	< .001	.982	.985	.050	.052	1.698
Comparison	*N*	*Δχ*^*2*^	*df*	*χ*^*2*^*/df*	*p*	*ΔCFI*				
b) vs a)	758	27.405	48	.571	.993	.006				
c) vs a)	758	130.53	110	1.187	.088	-.005				

CFI = Comparative Fit Index; TLI = Tucker Lewis Index; RMSEA = Root Mean Square Error of Approximation; SRMR = Standardized Root Mean square Residuals; WRMR = Weighted Root Mean square Residuals.

For the MI analysis for gender, item 11 in one group had an empty response category (5), so for this item categories 4 and 5 were merged. Separate CFAs showed good fit of the bifactor model for both genders. Configural invariance showed good robust fit indexes. Metric and scalar invariance showed slightly lower fit indexes but were still supported against configural invariance as the *ΔCFI* criterion was satisfied and normed chi-squares were close to 2. Interestingly, while there were no differences in latent means between genders for the general factor and the Inhibitory IU group factor, the Prospective IU group factor was significantly higher among men: 1.09 (*SE* = .144, *z* = 6.710, *p* < .001). A gender difference in the latent mean of the Prospective IU group factor provides further indication that it may be problematic. Importantly, the presence of full scalar invariance enables practitioners to make inferences on composite scores like test scores [85].

For the MI with respect to age, three age groups were defined: the first group contained people between 18–25 years of age, the second group between 26–45 years of age, and the last contained people 46 and older. Since in some groups the response category 5 was empty for items 10 and 11, categories 4 and 5 were merged for these items. As can be seen in Table 7, MI appears to be essentially satisfied up to the scalar level, hence there appears to be no substantial change in the factor structure of the IUS-R while describing IU across the adult lifespan. Please note that p = .088 for the last scaled chi square test of Table 7 is compatible with the presence of a small correlation between age and the IUS-R score.

### Temporal stability and longitudinal measurement invariance

For the undergraduate sample, Pearson’s correlation coefficient between the total scores at one month was *r* = .74 (95% *CI* = [.665, .807], *t(154)* = 13.827, *p* < .001) and the ICC coefficient was .74 (95% *CI* = [.656, .801], *F(155*, *156)* = 6.600, *p* < .001), suggesting good test-retest reliability. For MI (Table 8), since the response category 5 was empty for items 1, 2, 7 and 12 in at least one of the two temporal positions, categories 4 and 5 were merged for these items. The bifactor model was fitted to both test and retest conditions resulting in good robust fit indexes. Configural invariance also showed good fit indexes and the correlation between the general factor at the two administrations was .82 (*SE* = .028, *z* = 28.755, *p* < .001). Metric invariance showed good fit indexes as well, with a correlation of .81 (*SE* = .028, *z* = 29.428, *p* < .001). Scalar invariance showed robust fit indexes too, with again a correlation of .81 (*SE* = .028, *z* = 29.423, *p* < .001). There was a small decrease in the general latent factor mean of -.152 (*SE* = .068, *z* = -2.245, *p* = .025) between test and retest. Both metric and scalar invariances against configural invariance were supported.

**Table 8:** Fit statistics for the bifactor model tested for invariance across one-month test-retest (undergraduate sample).

Model	*N*	*χ*^*2*^	*df*	*χ*^*2*^*/df*	*p*	*CFI*	*TLI*	*RMSEA*	*SRMR*	*WRMR*
Test	156	76.127	42	1.813	< .01	.983	.973	.072	.053	.612
Re-test	156	58.319	42	1.389	.048	.992	.987	.050	.042	.500
a) Configural inv.	156	246.151	213	1.156	.059	.993	.991	.032	.051	.620
b) Metric inv.	156	265.523	237	1.120	.098	.994	.993	.028	.059	.737
c) Scalar inv.	156	302.332	278	1.088	.151	.995	.995	.024	.059	.768
Comparison	*N*	*Δχ*^*2*^	*df*	*χ*^*2*^*/df*	*p*	*ΔCFI*				
a) vs b)	156	9.325	24	.389	.997	.001				
a) vs c)	156	22.056	65	.339	1.00	.001				

CFI = Comparative Fit Index; TLI = Tucker Lewis Index; RMSEA = Root Mean Square Error of Approximation; SRMR = Standardized Root Mean square Residuals; WRMR = Weighted Root Mean square Residuals.

In order to extend the results of the previous MI to the community sample, an MI was conducted comparing the community and undergraduate samples. Since the response category 5 was empty for items 2 and 12 in the undergraduate sample, categories 4 and 5 were merged for these two items. Results of MI (Table 9) show that the bifactor structure is the same in both the samples.

**Table 9:** Fit statistics for the bifactor model tested for invariance across samples (community vs undergraduates).

Model	*N*	*χ*^*2*^	*df*	*χ*^*2*^*/df*	*p*	*CFI*	*TLI*	*RMSEA*	*SRMR*	*WRMR*
Community	758	139.167	42	3.314	< .001	.988	.982	.055	.032	.792
Undergraduate	162	63.991	42	1.524	< .001	.990	.984	.057	.051	.572
a) Configural inv.	920	191.303	84	2.277	<-001	.990	.984	.053	.035	.977
b) Metric inv.	920	184.967	108	1.713	< .001	.993	.991	.039	.043	1.315
c) Scalar inv.	920	252.109	139	1.814	< .001	.989	.990	.042	.040	1.377
Comparison	*N*	*Δχ*^*2*^	*df*	*χ*^*2*^*/df*	*p*	*ΔCFI*				
b) vs a)	920	20.837	24	.868	.64	.003				
c) vs a)	920	63.111	55	1.147	.21	-.001				

CFI = Comparative Fit Index; TLI = Tucker Lewis Index; RMSEA = Root Mean Square Error of Approximation; SRMR = Standardized Root Mean square Residuals; WRMR = Weighted Root Mean square Residuals.

## Discussion

IU is a trans-diagnostic factor that has been implicated in a wide range of psychological disorders and the utility of the IUS-12 in trans-diagnostic research and clinical practice is acknowledged. Walker et al. [53] adapted the IUS-12 to measure IU across the lifespan; importantly, their version of the test, i.e. the IUS-R, has proven to be a reliable and valid measure of IU in different samples [53–57], as well as in Italian undergraduates [58]. Beyond providing increasing evidence about the adequacy of the Italian IUS-R as a measure of IU, the current study addressed a number of yet unexplored or only partially explored issues which we will address in turn. Overall, the main results are in line with the expected results given in previous studies and further expand knowledge about the assessment of IU.

With respect to the factor structure of the questionnaire, results from the CFAs on the community sample suggested that the correlated two-factor model showed adequate fit indexes and might be a reasonable representation of the IU construct. However, the strength of the estimated correlation between the latent Prospective IU and Inhibitory IU domains (or between the predicted factor scores) suggested that there might be an unexpectedly high shared variance. Indeed, the application of a bifactor model as a tool to investigate the dimensionality of the scale and the strength of the group factors suggested that the Italian IUS-R is essentially a unidimensional scale. Such a result is supported by the presence of a strong common general factor in the bifactor model, and by the evaluation of composite reliability based on the bifactor solution. The general IU factor accounted for the 87% of variance in the IUS-R scores, consistent with, but slightly higher than the findings by Hale et al. (80%), Lauriola et al. (75%), and Shihata et al. (80% among undergraduates and 86% among clinical participants) [31, 42, 43]. Thus, there is increasing evidence across studies indicating that the total score is a good indicator of the general factor. Inspection of the standardized loadings across these three studies and the current study lead to some interesting similarities and differences.

First, a strong general factor was found in all samples. Loadings were generally comparable to those found in previous studies, with the only exception of items 3 and 4, which were lower in both Italian studies when compared to those in the studies by Hale et al. [31] and Shihata et al [43]. Interestingly, the content of these items explicitly refers to beliefs about the utility of planning under uncertain circumstances. Economics and business literature classifies Italy among the “strong uncertainty avoidance cultures” [86, 87]. Further, recent literature indicates that Italian people are likely to attribute a negative meaning to uncertainty and to be less confident about their own ability to solve problems and to manage uncertain events [88]. Consequently, Italian respondents may not fully endorse items about planning even when they are generally high in IU, perhaps explaining why lower loadings for these items have been observed in both the Italian studies. This supports the notion that cross-cultural differences may occur in the interpretation of (intolerance of) uncertainty.

Second, the Prospective IU domain has proved to be unreliable across all studies employing non clinical participants (in the clinical sample enrolled by Shihata et al. [43], the Prospective IU group factor even had to be removed in order to obtain an admissible bifactor model, consisting of a general IU factor and one group factor, inhibitory IU). The standardized loadings observed in the present study support the notion of a slightly stronger group factor than those reported by the other three studies [31, 42, 43] in undergraduate samples. The community sample and the different item phrasing of the IUS-R could have contributed to this result. However, none of the studies have retrieved robust group factors. The present study confirmed that some items on the group factor Prospective IU are particularly problematic. In particular, items 1 and 2 showed negative loadings, thus loading in the opposite directions to the other items on the factor. Although these items are coherent with the general factor representing IU, the part that does not load on the general factor is not coherent with the group factor. Negative loadings for item 1 were also observed also by Hale et al. [31] and Lauriola et al [42] but not Shihata et al. [43]. Both items refer to experiencing distress (i.e. feeling upset, feeling frustrated) in the face of uncertainty rather than engaging in activities that increase certainty/reduce uncertainty as indicated by the other items. As a consequence, it may be worth considering whether these items should be considered part of the Prospective IU domain in future research, since they clearly do not refer to the propensity to actively seek information to reduce uncertainty, although they may be a valid part of the general factor. Interestingly, item 2 was the only item showing a very high loading (.94) in the undergraduate sample reported by Shihata et al. [43], whereas other items showed extremely low loadings, ranging from -.03 to .18, indicating that in their sample the group factor may be more characterized by upset rather than information seeking.

Finally, when a correlation between Prospective IU and Inhibitory IU was permitted within the bifactor model, most of the items in the Prospective IU domain had negative loadings (which also resulted in a negative correlation between the latent group factors). Such a result suggests that, notwithstanding the fact that both Prospective IU and Inhibitory IU contribute to the IU construct, their specific content goes in opposite directions. This finding may be reasonable, given the original definition of the two dimensions: Prospective IU refers indeed to a strong desire for predictability, which drives individuals to collect as much information as possible in order to increase certainty/reduce uncertainty. In opposition, Inhibitory IU reflects “behavioural paralysis”, which might be intended as a sense of being stuck and unable to respond in the face of uncertainty [24, 30]. In other words, these two components represent conceptually opposite coping strategies employed to manage uncertainty, the former relying on active engagement and the latter on under-engagement [24].

Taken together, the results from this study indicate that the Prospective IU group factor is extremely unreliable and may collect unusual item behavior and distortions. Any further revision of the assessment of IU may require a more theory driven approach to adequately assess any group factors that may underlie the overall construct. In conclusion, our results support the presence of a strong and reliable general factor underlying the IUS-R items and provide good evidence of unidimensionality thus highlighting that the IUS-R total score is a reliable representation of the IU construct. Such a finding is conceptually sound and in line with the trans-diagnostic nature of IU, since unidimensional constructs are more likely than multidimensional ones to be invariant in terms of both form and function across individuals with different clinical phenotypes [31]. Some research has suggested that Prospective and Inhibitory IU may have differential links with specific disorders. For example, Prospective IU might drive several dysfunctional approach behaviors typical of GAD (i.e., worry) and OCD (i.e., compulsions), which are performed to reduce uncertainty/increase certainty; on the opposite, inhibitory IU would be more involved in disorders relying on avoidance behaviors to reduce exposure to uncertainty, as for example panic disorder, social anxiety, and depression [33, 34, 40]. However, findings from other studies [55, 89, 90] found that the two IU components were similarly associated with different symptom dimensions, consistent with the notion that people with high levels of IU might endorse both approach- and avoidance-oriented coping strategies to manage uncertainty and related distress but perhaps use them differentially in different situations. Consequently, the general trait IU is likely to have higher trans-diagnostic predictive utility than Prospective and Inhibitory IU separately [43].

As all the previous results are based on the application of a bifactor model, it should be noted that concern has been raised on its interpretation as structural and substantive models to describe scales [45]. Indeed, it has been argued that they might not be good representations of psychological phenomena, and that they might show superior fit performances only because they overfit data or are favored by fit statistics [46, 91]. In particular, some (but not all) of their better fit might derive from an ability to perform as “garbage collectors” for implausible response patterns, even though the data for most of the sample might actually be modeled with a unidimensional structure [44]. Although it appears that in general this issue should not arise between bifactor and correlated-factor models, but rather between bifactor and higher-order models [47], it is cautionary to not interpret them as substantive models. Notwithstanding these issues, they may still be good candidates to analyze the psychometric properties of test scores and of subscales based on group factors [48, 50]. In particular, they are helpful to investigate the reliability of the total score as a “univocal indicator of the latent variable, despite of the multidimensionality” ([49], p.232).

In the current study the bifactor model was used with this rationale, and it can be argued that the current version of the IUS-R has a reliable total score based on a unidimensional general factor, but that neither group factor achieves acceptable reliability. Practitioners who want to use the Inhibitory IU group factor as a separate scale (some group loadings were reasonably high thus indicating some degree of reliability) should consider that only about the 40% of the variance on the Inhibitory IU group factor is actually factor specific. The Prospective IU factor appears to be ill-conditioned (either by some items or by a theoretical concept that goes in the opposite direction of Inhibitory IU factor), hence it might not be advisable to use it as an independent subscale. Further refinements of the items or of the theoretical construct might enable the construction of a more reliable Prospective IU subscale. In such a case it would be reasonable that the correlated two-factor model (or a second order model) could be the best representation of the IU construct. Since, as previously outlined, Prospective and Inhibitory IU subscales might have differential relationships with clinically relevant constructs and specific measures of psychopathology [92], further analyses or indeed revision are needed to develop their reliabilities and relationships to the general construct. However, if the strong correlation between these two subscales is only a representation of their common relation with IU, it might be difficult to consistently find differential relationships with third variables. As an example, predicted factor scores on the two specific scales showed weak or non-significant correlations with other variables.

Analysis of correlations between the IUS-R total score with age and years of education provided results in line with our hypotheses. There were no associations between IUS-R total score and education, and only a weak correlation with age was observed. This also supports previous findings indicating that the IUS-R can be used with people of different ages and educational levels [53, 54]. The reasons for the decrease with age (as well as the non-significant trend in the associated MI analyses) are potentially an artefact, but may also be developmental as people gain experience and life unfolds with age; either uncertainty decreases, people’s intolerance of it does, or indeed both. Overall, demographic characteristics do not seem to affect the scores of the Italian IUS-R, which can be therefore administered in a wide range of samples.

As to convergent validity, current findings support both the established association between IU and GAD-related features (i.e., NPO and worry) [93] as well as the putative trans-diagnostic nature of IU where relationships with a variety of emotional disorders (such as GAD, social anxiety, panic disorder, depression, OCD) have been documented [18–20]. Medium-high correlations with NPOQ, OBQ-87 tolerance of uncertainty subscale, PSWQ, and SPS were observed, whereas correlations with OCI-R, BAI, BDI-II, and DASS-21 were weaker, though significant. Similar magnitudes are reported in the literature assessing associations between IU and these constructs in non-clinical samples, despite different studies using different questionnaires [26, 39, 40]. Furthermore, the strong association with the OBQ-87 tolerance of uncertainty subscale suggests that the IUS-R has adequate convergent validity [35]. Partial correlations between the IUS-R and the OBQ-87 tolerance of uncertainty subscale remained significant after controlling for the NPOQ and the PSWQ; the same occurred between the IUS-R and the NPOQ, whereas the association between the IUS-R and the PSWQ was no longer significant after controlling for the other measures. The particularly strong association between IU and NPO is in line with recent evidence suggesting that NPO might be better conceptualized as a facet of IU rather than as a distinct construct [93].

In addition to the previous considerations on the factor structure, we considered gender and age effects through MGCFA to investigate MI. In particular, scalar invariance of the bifactor model across gender was supported, as previously observed by Hale et al. [31]. In addition, MI was shown across three age groups. As a consequence, the IUS-R total score can be used to make inferences across genders, supplementing and extending findings from undergraduate samples [39, 58], and across the adult lifespan, thus supporting the rationale for which the IUS-R was developed [53]. Future research should consider further expanding these findings to children and adolescents. However, those wishing to use the subscales should be aware that Prospective IU might be heavily unreliable and that the associated total score might not be a reliable measure of the latent factor but only of the IU construct.

Finally, good one-month test-retest reliability in the undergraduate sample was observed in line with previous findings reported by Khawaja and Yu [36] on a similar group and consistent with the notion of IU as a dispositional, relatively stable feature [1, 16, 94]. To the authors’ knowledge, the current study is the first attempt to assess MI of the IU in a longitudinal study; the stability of the bifactor structure over time with a strong general factor was confirmed, further supporting the reliability of the IUS-R. In addition, this result can to a certain degree be extended to the community sample since MI of the bifactor structure was showed to hold when comparing community and undergraduate samples.

As to the main limitations of study, there are several points that require comment. First, the size of the undergraduate sample was relatively small and may not lead to stable solutions. Nevertheless, it should be noted that although the ratio of sample size to number of parameters [95] is not generally considered adequate to estimate the bifactor model and MI, it could be argued that with loadings of .5 or above and at least 8 indicators (requirements met here) the minimum required size is of about 160 observations [96]. Of course, results must be interpreted with caution and further studies are required to provide more reliable estimates. In addition, despite the evidence that the bifactor structure is the same in both undergraduates and community individuals, we did not directly test longitudinal MI in the community sample. Therefore, future studies investigating the temporal stability of the IUS-R in a non-student sample would be encouraged. Second, since the community sample was obtained by merging different samples collected in different waves, some of them did not complete all questionnaires which may have affected the results on convergent validity as they represent different subsamples or varying sizes. Third, despite the correlational data suggesting that the IUS-R scores are essentially insensitive to differences in educational level, MI across educational levels groups was not formally tested. Fourth, a measure of standard personality traits was not administered; similarly, we did not include measures assessing constructs theoretically unrelated to IU (e.g., sensation-seeking). Including such measures would have better situated our findings within a broader context as well as bolstered evidence for discriminant validity; consequently, the inclusion of such measures in future studies is desirable. Fifth, the MI of the Italian IUS-R factor model across non-clinical and clinical samples was not examined. This is an important issue, as results from community individuals may not generalize to patients with emotional disorders, although a very recent study reported that a bifactor model with a robust general factor best described IU in treatment-seeking individuals [43]. Finally, this was a correlational study with other self-report measures. Studies that use other indicators such as behavioral correlates of IU or real time responses to uncertainty would provide stronger evidence for the validity of the IUS-R. Moreover, the responsiveness of the IUS-R was not tested. We believe that data demonstrating sensitivity to change are required by the presence of evidences showing that IU is a trans-diagnostic and “trans-therapy” change process [22], and so changes in IU are correlated with or mediate treatment outcome (i.e. symptom reduction) in psychological interventions for emotional disorders [22, 97].

Despite these limitations, the coherence of the results suggests that the use of the Italian IUS-R in both research and clinical practice is warranted and suitable for people in a range of settings. Furthermore, the recommended use of the total score is fully consistent with recent literature and provides further support to the notion of IU as a trans-diagnostic factor [31]. Last but not least, the current findings suggest that future research on the measurement of IU through the IUS-12 and the IUS-R could focus on the refinement of item content from a conceptual standpoint, and on item phrasing, also taking into account the potential role played by cross-cultural differences in the interpretation and meaning of uncertainty. As a unidimensional scale, the Italian IUS-R seems fit for purpose but the Prospective IU domain (in particular) appears to require further work.

## Supporting information

S1 File The Italian IUS-R. (PDF)

S2 File Community sample dataset. (CSV)

S3 File Undergraduate sample dataset. (CSV)
