# Supplementary material for: Seeking certainty about Intolerance of Uncertainty: Addressing old and new issues through the Intolerance of Uncertainty Scale-Revised
Source: PLoS One. 2019 Feb 11;14(2):e0211929. doi: 10.1371/journal.pone.0211929 (PMC6370219; doi:10.1371/journal.pone.0211929)
Supplement: S1 File — (PDF) [file pone.0211929.s001.pdf]

IUS-R

ISTRUZIONI: Di seguito troverà una serie di affermazioni. La preghiamo di leggere attentamente ciascuna affermazione e di cerchiare quella che meglio la descrive.

1	Quando le cose accadono improvvisamente mi agito molto	Per niente d'accordo	Un po' d'accordo	Moderatamente d'accordo	Molto d'accordo	Completamente d'accordo
2	Le cose che non conosco mi infastidiscono	Per niente d'accordo	Un po' d'accordo	Moderatamente d'accordo	Molto d'accordo	Completamente d'accordo
3	Le persone dovrebbero sempre pensare a cosa potrebbe accadere. Questo farà sì che non accadano cose brutte	Per niente d'accordo	Un po' d'accordo	Moderatamente d'accordo	Molto d'accordo	Completamente d'accordo
4	Anche quando pianifichi molto bene le cose, un piccolo dettaglio può rovinare tutto	Per niente d'accordo	Un po' d'accordo	Moderatamente d'accordo	Molto d'accordo	Completamente d'accordo
5	Voglio sempre sapere cosa mi accadrà in futuro	Per niente d'accordo	Un po' d'accordo	Moderatamente d'accordo	Molto d'accordo	Completamente d'accordo
6	Non sopporto quando le cose accadono improvvisamente	Per niente d'accordo	Un po' d'accordo	Moderatamente d'accordo	Molto d'accordo	Completamente d'accordo
7	Dovrei essere sempre preparato prima che accada qualcosa	Per niente d'accordo	Un po' d'accordo	Moderatamente d'accordo	Molto d'accordo	Completamente d'accordo
8	Sentirmi incerto mi blocca nel fare la maggior parte delle cose	Per niente d'accordo	Un po' d'accordo	Moderatamente d'accordo	Molto d'accordo	Completamente d'accordo
9	Quando non sono certo su cosa fare rimango paralizzato	Per niente d'accordo	Un po' d'accordo	Moderatamente d'accordo	Molto d'accordo	Completamente d'accordo
10	Quando non so cosa accadrà, non riesco a fare le cose molto bene	Per niente d'accordo	Un po' d'accordo	Moderatamente d'accordo	Molto d'accordo	Completamente d'accordo
11	Anche la più piccola preoccupazione può bloccarmi nel fare le cose	Per niente d'accordo	Un po' d'accordo	Moderatamente d'accordo	Molto d'accordo	Completamente d'accordo
12	Devo stare lontano da tutte le cose di cui non sono certo	Per niente d'accordo	Un po' d'accordo	Moderatamente d'accordo	Molto d'accordo	Completamente d'accordo
